# Does the association between adherence to statin medications and mortality depend on measurement approach? A retrospective cohort study

**DOI:** 10.1186/s12874-017-0339-z

**Published:** 2017-04-20

**Authors:** Mhd. Wasem Alsabbagh, Dean Eurich, Lisa M. Lix, Thomas W. Wilson, David F. Blackburn

**Affiliations:** 1 School of Pharmacy, University of Waterloo, 10A Victoria St. S., Kitchener, ON N2G 1C5 Canada; 2 Department of Public Health Sciences, School of Public Health, University of Alberta, Edmonton, AB Canada; 3 Department of Community Health Sciences, Faculty of Medicine, University of Manitoba, Winnipeg, MB Canada; 4 Department of Medicine, Royal University Hospital, Saskatoon Health Region, Saskatoon, SK Canada; 5 College of Pharmacy and Nutrition, University of Saskatchewan, Saskatoon, SK Canada

**Keywords:** Compliance/adherence, Mortality, Treatment, Lipids and cholesterol, Secondary prevention

## Abstract

**Background:**

The aim of this study was to examine the relationship between mortality and statin adherence using two different approaches to adherence measurement (summary versus repeated-measures).

**Methods:**

A retrospective cohort study was conducted using administrative data from Saskatchewan, Canada between 1994 and 2008. Eligible individuals received a prescription for a statin following hospitalization for acute coronary syndrome (ACS). Adherence was measured using proportion of days covered (PDC) expressed either as: 1) a fixed summary measure, or 2) as a repeatedly measured covariate. Multivariable Cox-proportional hazards models were used to estimate the association between adherence and mortality.

**Results:**

Among 9,051 individuals, optimal adherence (≥80%) modeled with a fixed summary measure was not associated with mortality benefits (adjusted HR 0.97, 95% CI 0.86 to 1.09, *p* = 0.60). In contrast, repeated-measures approach resulted in a significant 25% reduction in the risk of death (adjusted HR 0.75, 95% CI 0.67 to 0.85, *p* < 0.01).

**Conclusions:**

Unlike the summary measure, the repeated measures approach produces a significant reduction of all-cause mortality with optimal adherence. This effect may be a result of the repeated measures approach being more sensitive, or more prone to survival bias. Our findings clearly demonstrate the need to undertake (and report) multiple approaches when assessing the benefits of medication adherence.

## Background

Observational studies using health-administrative databases have reported low mortality rates among individuals exhibiting high adherence to statin medications (HMG Co-A reductase inhibitors) [1]. However, these studies have produced highly variable estimates of benefit. Depending on the study, individuals exhibiting high adherence have been associated with 20%, [2] 50%, [3, 4] or even 81% [5] lower risks of death. An important source of variability may be the approach used to measure adherence, even if the adherence data source is the same.

In studies using electronic refill databases, adherence is often measured by the ‘medication possession ratio’ (MPR) or the ‘proportion of days covered’ (PDC). This approach estimates the percentage of days during a defined observation period where medication was available for consumption based on the total quantity obtained from pharmacy refills [6]. In descriptive studies, adherence is typically expressed as a single measure summarizing the entire observation period often lasting 1 year or more [7–9]. Although the summary measure of adherence offers a simple and straightforward approach to represent the entire period of follow-up, it does not account for the possibility that adherence may change during this period.

Medication adherence can also be measured repeatedly using defined intervals within a period of follow-up and treated as a time-dependent variable [10, 11]. This measurement method may have advantages over the summary measure because it is more sensitive to changes in adherence. For example, a summary adherence measure of 58% calculated over a 1-year period could actually reflect an individual with 16% adherence during the first 6 months and 100% adherence in the last 6 months of observation. It has been suggested that the repeated-measures approach is superior to the summary approach for identifying associations between adherence and mortality [11–13]. However, we can find no empirical data or theoretical paradigm to support this claim. No studies have investigated the impact of measurement strategy on the estimates of benefit of statin adherence. Thus, our purpose was to compare the estimated impact of statin adherence on mortality using two measurement approaches, a fixed summary measure versus repeated-measures, for a cohort of individuals with acute coronary syndrome (ACS).

## Methods

### Data source

Administrative data maintained by the Saskatchewan Ministry of Health were used for this study. Saskatchewan Ministry of Health databases contain comprehensive data and have been used previously to produce high quality pharmacoepidemiological studies [14–18]. Specifically, we used information from the population registry, prescription drug file (pharmacy dispensations), physician claims, and hospital services databases. The Saskatchewan Ministry of Health data covers almost 99% of the province’s residents for both physician and hospital services. The only exceptions are federal prisons inmates, members of the armed forces, and the Royal Canadian Mounted Police, who are recipients of the federal government’s health benefits. On the other hand, the prescription drug database captures medication dispensations for 90% of the provincial population; it excludes individuals who receive federal prescription coverage such as the First Nations (aboriginal) population. Information on medications available “over-the-counter” or excluded from the provincial drug formulary were not available in this study.

### Cohort

The cohort included individuals at least 30 years of age who received at least one dispensation for a statin medication within 90 days [2, 19] of hospital discharge and for whom the responsible/primary diagnosis in the hospital record was ACS. This included myocardial infarction (MI) and unstable angina (UA). All individuals who met the cohort inclusion criteria between January 1^st^, 1994 and December 31^st^, 2008 were retained. Individuals were required to have continuous beneficiary status for 1825 days (i.e., 5 years) before the index hospitalization. Additionally, in order to offer the opportunity to exhibit adherence or non-adherence behaviour, individuals had to survive and maintain provincial beneficiary status for at least 102 days after their first statin dispensation. Individuals were excluded if they underwent a revascularization procedure without a diagnosis of MI or UA, could not be followed for at least 102 days, or received any statin medication within 365 days prior to the index hospitalization [20]. The codes used to identify MI and UA conditions (Appendix 1) were shown to have positive predictive, sensitivity, and specificity estimates of 85 to 98% [21–26]. For individuals with several eligible hospitalizations, the earliest hospital discharge date for ACS was deemed the index date.

### Adherence

**Fig. 1:**
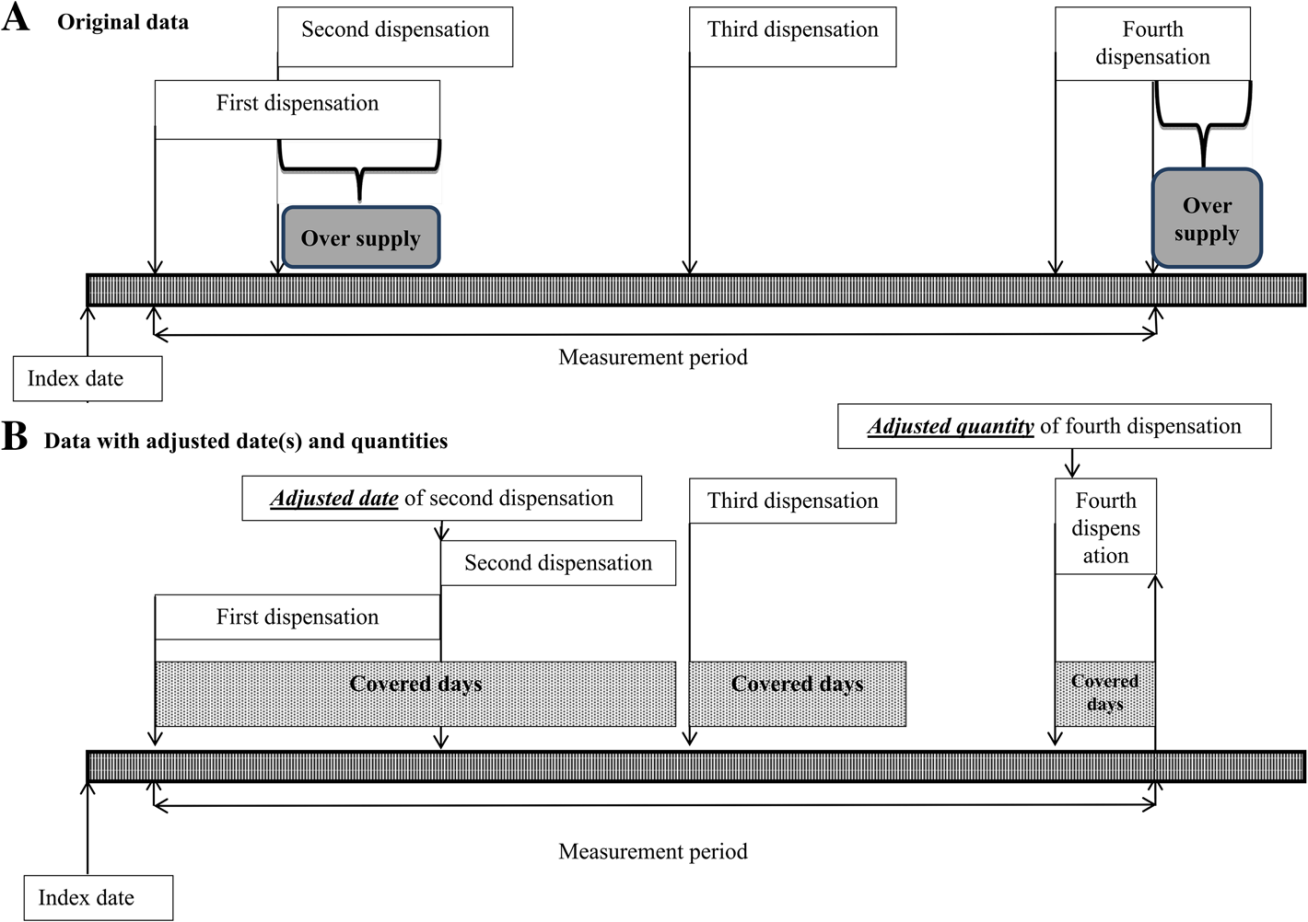
Adjustment of the adherence measure (proportion of days covered) to prevent overestimation from early refills or refills extending beyond measurement period represented on days’ time scale (shadowed panels) as original data **a** and data with adjusted date(s) and quantities **b**

Adherence was measured from the first statin dispensation date until death, provincial health coverage termination, or end of the study period (i.e., December 31, 2008). The PDC method was used to calculate adherence [27–29] with an adjustment to prevent overestimation. Specifically, each statin dispensation was assigned a ‘completion date’ corresponding to the number of medication doses supplied [30]. If a subsequent dispensation was obtained early, the new supply was not applied until the previous ‘completion date’ plus 1 day. Also, any excess supply of medication extending beyond the last follow-up day was removed from the calculation (Fig. 1) [30]. Similar to other studies, 80% level of adherence or higher was considered optimal adherence [31, 32] and switching between statins was allowed. Additionally, we removed any days of hospitalization during the observation period from PDC calculation because medications dispensed to inpatients are not included in the prescription drug database [33].

The assessment of adherence was applied in two ways. In method “A”, a single summary measure of adherence was calculated between the date of the first dispensation and the date of death, provincial health coverage termination, or end of the study period (December 31, 2008). In method “B”, the same period of follow-up was divided into 3-month intervals (i.e., 102 days) where adherence was measured in each. Unused supplies from a previous interval were applied to the subsequent interval to prevent underestimation. The 102 days interval was chosen because, in Saskatchewan, prescriptions are usually filled in 1 month supply quantity (34 days) [34].

### Analysis procedure

The association between statin adherence and mortality was described using Kaplan-Meier estimator and was assessed using a time-to-event analysis with multivariable Cox proportional-hazard regression models. The outcome was time to death, starting 102 days following the first statin dispensation. The model covariates included demographic, condition-related, therapy-related, patient-related, and health-system-related variables, as categorical variables (Appendix 2, Appendix 3 and Appendix 4), in addition to dichotomous adherence variable (i.e., PDC ≥80% versus PDC < 80%). For Method A, the summary measure of adherence was entered in the model as a fixed covariate (i.e. a covariate that does not differ in value over time), whereas for Method B, adherence was included in the model as a time-dependent repeated-measure covariate (i.e. a covariate that differs in value over time) assessed every 102 days.

We estimated the adjusted hazard ratios (HRs) with 95% confidence intervals (95% CIs) for adherent individuals (i.e. PDC ≥ 80%) compared with non-adherent individuals (i.e., PDC < 80%). The proportional hazards assumption was assessed visually using the log cumulative hazard (the “log-log”) plot and Schoenfeld residuals versus observed event time’s plot [35]. Multicollinearity amongst the non-adherence variables was examined by calculating the variance inflation factor (VIF); values greater than 10 were interpreted as representing substantial multicollinearity [36]. Baseline variables that demonstrated evidence of multicollinearity were excluded from the model.

In a sensitivity analysis, adherence was re-classified into three categories (instead of two): PDC 20%, 21–79%, and ≥80% to assess whether estimates of benefit were substantially affected. We used SAS 9.3 software (SAS Institute Inc., Cary, NC, USA) to perform all analyses.

## Results

**Fig. 2:**
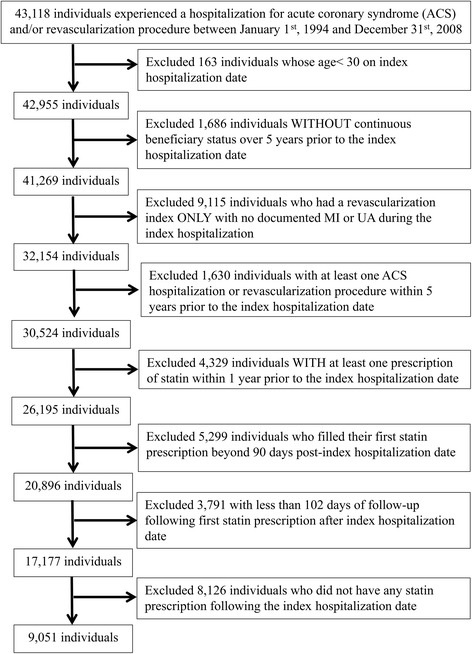
Flow chart for cohort selection

From 43,118 individuals who were hospitalized and had an ACS diagnosis and/or a coronary revascularization procedure in Saskatchewan between January 1^st^, 1994 and December 31^st^, 2008, a total of 9,051 individuals (21.0%) met all inclusion criteria (Fig. 2).

Among all individuals in the cohort, 69.2% (*n* = 6,260) were male and the mean age was 64.8 years (median = 66.0, standard deviation [SD] = 12.3). More than half of individuals (58.5%; *n* = 5,292) received a revascularization procedure during their ACS hospitalization. Additionally, roughly one third (36.7%, *n* = 3,325) had a diagnosis of hypertension in the year prior to the index date, and 13.6% (*n* = 1,232) had a diabetes diagnosis (Table 1). The mean follow-up time was 1,721 days (median 1,525.0, SD = 1,138.4) or 4.7 years. The mean PDC calculated over the entire follow-up period was 70.6% (median = 84.0%, SD = 31.9%), and the percentage of individuals achieving optimal adherence (i.e. ≥80%) was 54.6% (*n* = 4,939). The percentage of adherent individuals increased substantially over the study period from 40.7% in 1994 to 77.8% in 2008.

**Table 1:** Baseline characteristics

Characteristic		Non-Adherent^*^ (*n* = 4,112)	Adherent (*n* = 4,939)	Total (*n* = 9,051)	*p*-value^£^
		n (%)	n (%)	n (%)	
Age	mean (SD)	64.2 (12.6)	65.4 (12.0)	64.8 (12.3)	0.01^£^
	<55	1050 (25.5%)	1071 (21.7%)	2121 (23.4%)	0.30
	55–65	1036 (25.2%)	1311 (26.5%)	2347 (25.9%)	
	66–73	849 (20.6%)	1062 (21.5%)	1911 (21.1%)	
	≥74	1177 (28.6%)	1495 (30.3%)	2672 (29.5%)	
Male gender		2854 (69.4%)	3406 (69.0%)	6260 (69.2%)	0.91
Index year	1994–1997	329 (8.0%)	218 (4.3%)	547 (6.0%)	<0.01
	1998–2001	1141 (27.7%)	922 (18.7%)	2466 (22.8%)	
	2002–2005	1777 (43.2%)	2018 (41.0%)	4202 (41.9%)	
	2006–2007	865 (21.0%)	1781 (36%)	2135 (30.0%)	
Type of index diagnosis	ACS+ revascularization procedure	2098 (51.0%)	3149 (64.7%)	5292 (58.5%)	<0.01
	ACS only	2014 (49.0%)	1745 (35.3%)	3759 (41.5%)	
Duration (in days) of index hospitalization	mean (SD)	9.2 (8.5)	8.2 (9.9)	8.9 (9.0)	<0.01
	≥10 days	935 (22.7%)	1378 (27.9%)	2313 (25.6%)	<0.01
Time (in days) from index to statin prescription	mean (SD)	10.3 (21.4)	7.7 (18.7)	8.5 (19.6)	<0.01
	>1 day	1011 (24.6%)	894 (18.1%)	1905 (21.0%)	<0.01
At least one prescription in post-index year	BB	3449 (83.9%)	4262 (86.3%)	7711 (85.2%)	<0.01
	ACEI/ARB	3199 (77.8%)	7282 (86.7%)	7481 (82.7%)	<0.01
	CCB	890 (21.6%)	1078 (21.8%)	1968 (21.7%)	0.96
	diuretic	1389 (33.8%)	1865 (37.8%)	3254 (36.0%)	<0.01
	anticoagulants	586 (14.3%)	870 (17.6%)	1456 (16.1%)	<0.01
	antiplatelet	2228 (54.2%)	3108 (64.4%)	5408 (59.8%)	<0.01
	nitrates	2916 (70.9%)	3529 (71.5%)	6445 (71.2%)	0.92
	other lipid drugs	213 (5.2%)	187 (3.8%)	400 (4.4%)	<0.01
At least a statin prescription with 28 days’ supply (as an evidence of unit-of-use packaging)		174 (4.2%)	510 (10.3%)	684 (7.6%)	<0.01
High statin dose on first prescription post index^€^		2141 (52.1%)	3166 (64.1%)	5307 (58.6%)	<0.01
Atorvastatin on first prescription post index		2933 (59.4%)	2388 (58.1%)	5321 (58.8%)	0.17
>4 distinct non-statin medications received in post-index year		2397 (58.3%)	3302 (66.9%)	5699 (63.0%)	<0.01
Chronic disease score ≥4		404 (8.2%)	325 (7.9%)	729 (8.1%)	0.63
Diagnosis in pre-index year	DM	532 (12.9%)	700 (14.2%)	1232 (13.6%)	0.62
	HTN	1406 (34.2%)	1919 (38.9%)	3325 (36.7%)	0.01
Specialty of prescribing physician of the first statin prescription	GP	633 (15.4%)	579 (11.7%)	1212 (13.4%)	<0.01
	cardiologist	1937 (47.1%)	2673 (54.1%)	4610 (50.9%)	
	internist	984 (23.9%)	998 (20.2%)	1982 (21.9%)	
	cardiac surgeon	239 (5.8%)	384 (7.8%)	623 (6.9%)	
	other	319 (7.8%)	305 (6.2%)	624 (6.9%)	
≥5 physician’s visits in the first 3 months		3030 (73.7%)	3849 (77.9%)	6879 (76.0%)	<0.01
Any hospitalization in pre-index year		1011 (24.6%)	1109 (22.5%)	2120 (23.4%)	<0.01
Deprivation index quintile	missing	168 (4.1%)	161 (3.3%)	329 (3.6%)	-
	1 (most deprived)	871 (21.2%)	947 (19.2%)	1818 (20.1%)	0.01
	2	680 (16.5%)	789 (16.0%)	1469 (16.2%)	
	3	881 (21.4%)	1109 (22.5%)	1990 (22.0%)	
	4	779 (18.9%)	1006 (20.4%)	1785 (19.7%)	
	5 (least deprived)	733 (17.8%)	927 (18.8%)	1660 (18.3%)	

**Fig. 3:**
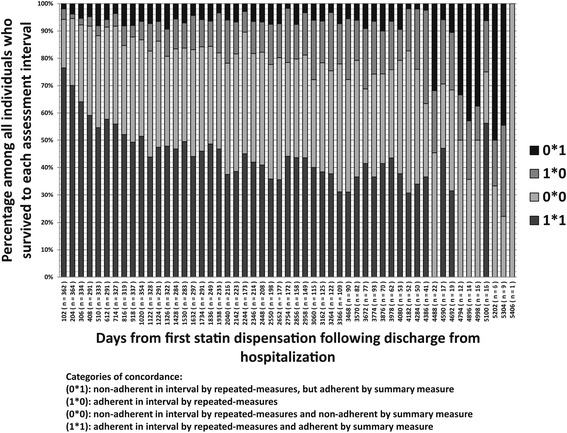
Concordance between two measures of statin adherence (summary measure and repeated-measures) among individuals with coronary heart disease

*Individuals with PDC of less than 80% were considered non-adherent. Abbreviations: *ACS* acute coronary syndrome, *ACEI*/*ARB* angiotensin converting enzyme-inhibitor/angiotensin receptor-blockers, *BB* beta-blockers, *CCBs* calcium channel blockers, *DM* diabetes mellitus, *GP* general practitioner, *HTN* hypertension, *PDC* proportion of days covered, *PROC* procedure of revascularization; €: High dose statin was defined as having rosuvastatin >5 mg, atorvastatin ≥20 mg, or simvastatin ≥40 mg; £: tests of differences for continuous variables were conducted using t-tests and for categorical variables were conducted using chi-square tests;

Adherence categorization by the fixed baseline summary measure was generally concordant with the repeated measures approach in the last completed measurement interval. The adherence category matched on both measures in 76.7% of individuals (median 80.2%, SD = 15.4%) However, the concordance between the two measures declined over time (Fig. 3). Non-concordance was most commonly observed as non-adherence by the fixed summary measure and optimal adherence using the repeated-measures approach. This may reflect the case when an individual starts therapy by being non-adherent early in the treatment period, and then becomes adherent afterward. Essentially, the fixed summary measure penalizes individuals for previous periods of non-adherence whereas the repeated measures approach does not consider past adherence patterns. In contrast, the percentage of cases of optimal adherence by the fixed summary measure but poor adherence by the repeated-measures was relatively infrequent and remained stable over time (Fig. 3).

**Fig. 4:**
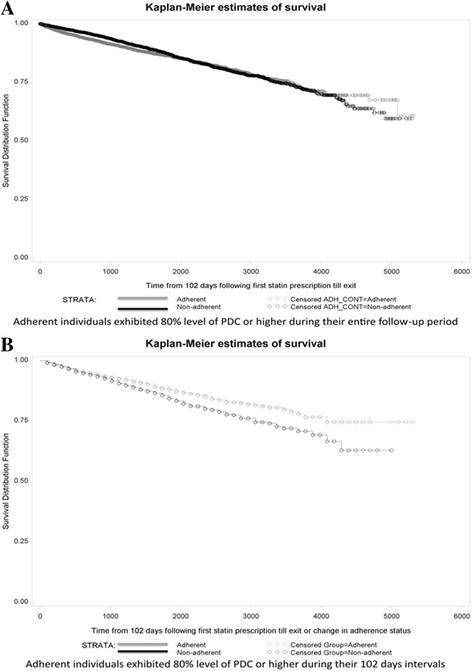
Kaplan-Meier estimates of survival among individuals classified using adherence summary measure (**a**) and adherence repeated-measures (**b**)

Using the fixed-summary measure of adherence, death occurred in 12.3% (*n* = 606) of adherent individuals (i.e.PDC ≥80%) compared to 14.6% (*n* = 600) of non-adherent individuals. This difference was not associated with a lower risk of death in the time-to-event analysis (Fig. 4) (crude HR 1.07, 95% CI 0.96 to 1.20, *p* = 0.25; adjusted HR 0.97, 95% CI 0.86 to 1.09, *p* = 0.60). Similar results were obtained when non-adherence was categorized as < 20% (i.e., rather than < 80%) in a sensitivity analysis (crude HR 0.91, 95% CI 0.77 to 1.08; *p* = 0.28; adjusted HR 0.96, 95% CI 0.80 to 1.14). This effect was identical in the 1899 individuals who entered the cohort prior to 2001 (crude HR 0.95, 95% CI 0.78 to 1.16; *p* = 0.62; adjusted HR 0.93, 95% CI 0.75 to 1.14, *p* = 0.48), and the 7,152 individuals who entered the cohort on 2001 and after (crude HR 1.09, 95% CI 0.95to 1.26; *p* = 0.23; adjusted HR 1.01, 95% CI 0.87 to 1.17, *p* = 0.91).

In contrast, optimal adherence measured as a time-dependent variable was clearly associated with a lower risk for death (Fig. 4) (crude HR 0.80, 95% CI 0.71 to 0.89, *p* < 0.01; adjusted HR 0.75, 95% CI 0.67 to 0.85, *p* < 0.01). Similar results were obtained when non-adherence was categorized as <20% (i.e., rather than < 80%) in a sensitivity analysis (data not shown). Additionally, almost indistinguishable results were obtained when the required time for filling statin prescription was reduced to 60 days or increased to 120,180, or 210 days. Similarly, when adherence was considered a continuous variable, it was not associated with mortality using the fixed-summary measure method (crude HR 1.05; 95% CI 0.88 to 1.25; *p* = 0.63 and adjusted HR 1.00; 95% CI 0.83 to 1.20, *p* = 0.97 for an increase of 1 unit of PDC), but it was strongly associated with mortality using the repeated-measures method (crude HR 0.65; 95% CI 0.56 to 0.75, *p* < 0.01 and adjusted HR 0.66; 95% CI 0.56 to 0.76, *p* < 0.01 for an increase of 1 unit of PDC).

We examined the impact of adherence during two distinct periods: the last follow-up interval (i.e. the last 102 days of the follow-up) and the first follow-up interval (i.e., the first 102 days of follow-up). Treating the adherence in the final interval as a fixed variable was significantly associated with mortality with a crude HR of 0.83, (95% CI 0.75 to 0.93, *p* < 0.01) and adjusted HR of 0.85 (95% CI 0.75 to 0.95, *p* < 0.01). On the other hand, considering only the first period to measure adherence did not yield a significant association with mortality with a crude HR of 0.95, 95% CI 0.84 to 1.09, *p* = 0.47, and adjusted HR of 0.88, 95% CI 0.77 to 1.01, *p* = 0.07.

In all cases, the proportionality assumption of the Cox model was met, and no collinearity was observed in included covariates.

## Discussion

We examined the association between statin adherence and the risk of death using two distinct adherence measures that have been used in previous studies [2]. The association was substantially impacted by the measurement approach despite an identical adherence metric (i.e., PDC) and threshold (i.e., ≥80%) for defining optimal adherence. Optimal statin adherence defined by the fixed summary measure was not associated with a beneficial effect on mortality (adjusted HR 0.97, 95% CI 0.86 to 1.09, *p* = 0.60). In contrast, optimal adherence to statins defined by a time-dependent variable was associated with a significantly lower risk of death (adjusted HR 0.75, 95% CI 0.67 to 0.85). Although medication non-adherence is widely understood as a public health epidemic, it should be recognized that the estimated benefits of optimal adherence are not robust to changes in measurement approaches.

It is well known that the format of an independent variable (time-dependent or fixed at baseline) can impact its statistical association with an outcome [37, 38]. In certain situations, the use of time-dependent variables is clearly appropriate. For example, consider a study examining the occurrence of bleeding due to anticoagulant use. Exposure to anticoagulants could be modeled as a time-dependent variable because bleeding risk increases promptly after exposure (from a reversible pharmacologic process) and resolves following discontinuation. In contrast, statin medications modulate the vascular atherosclerotic disease process. Thus, they would be expected to have a much slower onset of protection and sustained benefit following drug discontinuation. In fact, it could be argued that the duration of past adherence (estimated more effectively with a summary adherence measure of statin medications) is more important than recent adherence due to the requirement of statin exposure-time for modification of vascular atherosclerosis.

The reasons for such conflicting estimates on the association between statin adherence measurements are not entirely clear. Our study was carried out on the same cohort, over the same observation period, and accounted for identical confounders with the exception of adherence measurement. The repeated-measures approach appeared to take account of changes in adherence over time, especially situations where individuals improved their adherence behavior over time. Indeed, non-concordance between adherence measures was virtually always observed as patients assessed as non-adherent by the summary measure while adherent by the repeated measure. Low adherence at any point in an observation period will always penalize the final summary adherence assessment, whereas the repeated-measures approach adjusts estimates based on independent adherence measurements at 3-month intervals [39].

In a secondary analysis, optimal adherence during the final 3-month interval of follow-up was significantly associated with a lower risk of death. From a pharmacologic perspective, this association is difficult to justify due to the short period of statin exposure. However, it can be argued that this approach permitted the influence of survival bias [12, 13]. Healthy individuals with long-standing non-adherence may have had greater opportunity to exhibit optimal adherence in the latter part of the observation period using a repeated-measures approach. In this situation, high adherence in the last 3 months of follow-up may have been a marker rather than a causal determinant of good health. Although the repeated-measures approach undoubtedly captures more granular information about exposure, [37, 40] the relationship between *current* exposure and protection against life-threatening outcomes is not as clearly defined for cholesterol-lowering therapy. Moreover, it may be more vulnerable to bias or confounding factors that impact the outcome.

Our study identified a dramatic improvement in statin adherence over the past decade. This trend has been reported previously in other jurisdictions with statins and other medications also [41, 42]. Considering these trends, along with steady population decreases in coronary heart diseases event rates over time, [43] it is possible that the consequences of poor statin adherence may in fact be less dramatic in recent years. Although conflicting results from observational studies could be ideally resolved if randomized trial results were available, the nature of this phenomenon prevents rigorous examination using experimental design.

The most important strength of our study is that we performed both measurement approaches on the same cohort of individuals with the same characteristics that can affect adherence (such as socio-economic status, motivation, and attitudes). However, some limitations can be noted in this study. First, although PDC is a validated adherence measurement method, our adjustment to prevent overestimation is not validated, and may have affected our estimates. However, it is unlikely that this adjustment disadvantaged one of the methods only (i.e., the summary approach or the repeated-measures approach). Second, requiring individuals to fill a statin prescription within 90 days of their ACS hospitalization may have excluded individuals exhibiting non-adherence at the beginning of follow-up (primary non-adherence). If true, this could have weakened the association through a biased selection of individuals. Third, requiring individuals to survive for at least 102 days after their first statin prescription may have biased our inclusion to include patients with less severe heart disease. However, events occurring within the first 3 months after beginning statin therapy are likely not related to statin adherence levels. Fourth, having a record of statin dispensation does not certainly indicate that the medication was in fact consumed. Lastly, the choice of 102 days (3 months) to assess adherence in the repeated-measures method may have influenced the associations observed. However, shorter intervals would result in lower granularity of the measure and longer periods would result in lower sensitivity to periodic changes in adherence.

## Conclusions

Statin adherence after acute coronary syndromes has improved dramatically since the 1990s and is nearing 80% in recent years. Estimates for the benefits of statin adherence on mortality are significantly influenced by measurement methods used and a gold-standard approach cannot be established using conventional techniques. Further, estimates of population benefits of statin adherence may have been exaggerated due to the lack of verification with different approaches. Although surveillance of adherence and health outcomes should continue, estimates must be scrutinized using different measures until the most valid approach can be identified.

## Appendix 1

**Table 2:** The codes used to include eligible individuals in the cohort

Individual Selection Diagnoses from Hospital Services Database		
ICD-9^a^	ICD-10-CA	Description
410	I21–I22.xxx	myocardial infarction (MI)
411	I20.0xx and I24.xxx	unstable angina (UA)

^a^ICD-9 was used until March 31, 2001, when ICD-10-CA reporting started

ICD-9: Manual of the International Statistical Classification of Diseases, Injuries, And Causes Of Death, 9th revision. Geneva: The Organization; 1977

ICD-10-CA: International Statistical Classification of Diseases and Related Health Problems, tenth revision, Canada. Canadian Institute for Health Information; 2003

## Appendix 2

**Table 3:** Variables considered for the baseline adjustment

Variable category	Included variables
Demographic variables	• age at index date
	• gender
	• year of hospital discharge
Condition-related variables	• type of index diagnosis (ACS only, ACS plus revascularization)
	• duration of index hospitalization
	• number of days between index date and first dispensation of statin
Therapy-related factors	• filling at least one prescription for specific cardiovascular medication (s) during the first year post-index including (beta-blockers; angiotensin converting enzyme-inhibitor (ACEI) or angiotensin receptor-blockers (ARB); calcium channel blockers (CCBs); diuretics; anticoagulants; antiplatelet; nitrates; or other lipid drugs - yes/no for each) (Appendix 3)
	• filling at least one prescription with a quantity of 28 tablets as an evidence of unit-of-use packaging
	• receiving a high (versus low) statin dose on first prescription post index
	• the statin used on first prescription following the index date (atorvastatin versus others)
	• burden of medications defined as the total number of distinct medications’ therapeutic groups dispensed to the individual
Patient-related factors	• socio-economic status (SES) assessed by the deprivation index (DI) developed by *Pampalon* et al. This was identified by mapping individuals’ residential postal codes to geographic-level statistics for the Statistics Canada census.
	• comorbidities calculated by Deyo-adapted Charlson score method using hospitalizations data
	• specific comorbid conditions of diabetes, and hypertension reported in any physician claim or hospitalization in the year prior to the index date (Appendix 4)
Health system-related factors	• specialty of prescribing physician for the first statin dispensation (general practitioner (GP), cardiologist, general internist, cardiac surgeon, other)
	• number of physician billing claims in the first 3 months following the first statin dispensation
	• any prior hospitalization in the year prior to the index date.

## Appendix 3

**Table 4:** Medications included in the multivariable model

Medication category	Generic name of medications included	
Statin mediation (i.e., HMG-CoA reductase inhibitors)	Atorvastatin, atorvastatin/amlodipine combination, cerivastatin, fluvastatin, lovastatin, pravastatin, rosuvastatin, and simvastatin	
Angiotensin Converting Enzyme-Inhibitors (ACE-Inhibitors)	Benazepril, captopril, cilazapril, cilazapril/HCTZ combination, enalapril, enalapril/HCTZ combination, fosinopril, lisinopril lisinopril/HCTZ combination, perindopril, perindopril/indapamide combination, quinapril quinapril/HCTZ combination, ramipril, and trandolapril	
Angiotensin Receptor-Blockers (ARBs)	Candesartan, candesartan/HCTZ combination, eprosartan, eprosartan/HCTZ combination, irbesartan, irbesartan/HCTZ combination, losartan, losartan/HCTZ combination, olmesartan, Olemsartan/HCTZ combination, telmisartan, telmisartan/HCTZ combination, valsartan, and valsartan/HCTZ combination	
Beta Blockers	Acebutolol, atenolol, atenolol/chlorthalidone combination, labetolol, metoprolol, metoprolol/HCTZ combination, nadolol, oxprenolol, pindolol, pindolol/HCTZ combination, propranolol, propranolol/HCTZ combination, timolol, and timolol/HCTZ combination	
Calcium Channel Blockers (CCBs)	Dihydropyridine (DH-CCB)	Amlodipine, felodipine, nicardipine, and nifedipine long acting,
	Non-dihydropyridine (NDH-CCB)	diltiazem, and verapamil
Diuretics	Amiloride, amiloride/HCTZ, bumetanide, chlorthalidone, ethacrynic acid, furosemide, hydrochlorothiazide (HCTZ), indapamide, metolazone, spironolactone, spironolactone/HCTZ, triamterene, triamterene/HCTZ	
Anticoagulants	Acenocoumarol, dalteparin, enoxaparin, heparin, nadroparin, tinzaparin, warfarin	
Antiplatelet	ASA, clopidogrel, dipyridamole/ASA, pentoxifylline, sulfinpyrazone, ticlopidine	
Nitrates	Erythrityl tetranitrate, isosorbide dinitrate, isosorbide-5-mononitrate, nitroglycerin	
Other lipid drugs	Bezafibrate, cholestyramine, clofibrate, colestipol, ezetimibe, fenofibrate, gemfibrozil, niacin, probucol	

## Appendix 4

**Table 5:** Definitions of comorbid conditions included in the multivariable model

ICD-9^a^	ICD-10-CA	Description
250.x	E10 - E14.xxx	Diabetes & diabetes with complications
401–404.x	I10 to I15.xxx except when I11 is reported with I50 where’s the individual was considered to have heart failure	hypertension

^a^ICD-9 was used until March 31, 2001, when ICD-10-CA reporting started

ICD-9: Manual of the International Statistical Classification of Diseases, Injuries, and Causes Of Death, 9th revision. Geneva: The Organization; 1977

ICD-10-CA: International Statistical Classification of Diseases and Related Health Problems, tenth revision, Canada. Canadian Institute for Health Information; 2003

## Abbreviations

ACS: Acute coronary syndrome
CIs: Confidence intervals
HRs: Hazard ratios
MI: Myocardial infarction
MPR: Medication possession ratio
OTC: Over-the-counter
PDC: Proportion of days covered
UA: Unstable angina
VIF: Variance inflation factor
